# Vaccination demonstration zone successfully controls rabies in Guangxi Province, China

**DOI:** 10.1186/s12879-018-3301-8

**Published:** 2018-08-10

**Authors:** Xian-Kai Wei, Yi Xiong, Xiao-Ning Li, Min Zheng, Yan Pan, Xiao-Xia He, Jing-Jing Liang, Cheng Liu, Yi-Zhi Zhong, Lian-Bin Zou, Lie-Feng Zheng, Jian-Gang Guo, Chang-Ting Li, Sheng-Bin Huang, Jia-Zhong Gan, Zhen-Mu Meng, Jian Yang, Hai-Bo Tang, Qi Liu, Ting Rong Luo

**Affiliations:** 1 State Key Laboratory for Conservation and Utilization of Subtropical Agro-Bioresourses, Guangxi University, 100# Daxue Road, Nanning, 530004 Guangxi China; 2 Laboratory of Veterinary Microbiology and Animal Infectious Diseases, College of Animal Sciences and Veterinary Medicine, Guangxi University, 100# Daxue Road, Nanning, 530004 Guangxi China; 3 Guangxi Center for Animal Disease Control and Prevention, Nanning, 530001 Guangxi China; 4 Guangxi Veterinary Research Institute, Nanning, 530001 Guangxi China; 5 Yulin Center for Animal Disease Control and Prevention, Yulin, 537000 Guangxi China; 6 Baise Center for Animal Disease Control and Prevention, Baise, 533000 Guangxi China

**Keywords:** Rabies, Vaccination demonstration program, Vaccination coverage rate, Seroconversion rate

## Abstract

**Background:**

Guangxi is the province most seriously affected by rabies virus (RABV) in China. Those most affected by RABV each year are people in rural areas, where dogs are the main cause of human infection with the virus.

**Methods:**

In this study, we established a rabies vaccination demonstration program that included eradication, core, and peripheral areas. This program was implemented for 9 years and comprised three stages: 12 counties in the first stage (2008–2010), 21 counties in the second stage (2011–2013), and then extending to all counties of Guangxi Province in the third stage (2014–2016). The program included a dog vaccination campaign, surveillance of clinically healthy dogs who may be potential RABV carriers, monitoring anti-RABV antibody titers in vaccinated dogs, and compiling and reporting statistics of human rabies cases.

**Results:**

The target effectiveness was achieved in the eradication, core, and peripheral areas in all three stages. The vaccination demonstration program successfully promoted RABV vaccination of domestic dogs throughout Guangxi Province by drawing upon the experience gained at key points. Compared with a vaccination coverage rate of 39.42–46.85% in Guangxi Province overall during 2003–2007, this rate gradually increased to 48.98–52.67% in 2008–2010, 60.24–69.67% in 2011–2013, and 70.09–71.53% in 2014–2016, thereby meeting World Health Organization requirements. The total cases of human rabies in the province decreased from 602 in 2004 to 41 cases in 2017.

**Conclusions:**

The present pilot vaccination program obviously increased the rabies vaccination and seroconversion rates, and effectively reduced the spread of rabies from dogs to humans as well as the number of human rabies cases, thus successfully controlling rabies in Guangxi.

**Electronic supplementary material:**

The online version of this article (10.1186/s12879-018-3301-8) contains supplementary material, which is available to authorized users.

## Background

Rabies is a neglected zoonosis with a 100% fatality rate [1]. However, the disease is completely preventable through timely intervention with postexposure rabies vaccine and immunoglobulin. China is one of the countries that has experienced serious impact from this disease, with the number of human rabies cases ranking second highest in the world after India [2, 3]. Guangxi Province (Additional file 1: Figure S1) is the most severe epidemic region of human rabies in China [4–6].

According to the global framework for eliminating rabies, China has responded to the target of zero human rabies deaths by 2030 by scaling up their efforts to consign rabies to the history books [1]. Epidemiological surveys and phylogenetic analyses have indicated that elimination of rabies among domestic dogs in rural areas is critical for the control of human rabies in China [2].

It is understandable that the medical sector emphasizes prevention of human rabies through postexposure prophylaxis (PEP). However, this approach may lead to neglecting the problem of the fountainhead, as dogs are a principal factor in the spread of rabies to humans. A PEP-based approach to prevention can impede progress toward instituting large-scale dog vaccination campaigns [7]. This is true even in upper middle-income countries, which have clear capability to implement mass dog vaccination but have failed to effectively carry out such programs. These countries still have a high burden of human deaths and therefore experience continued escalation of the demand for PEP, with costs amounting to hundreds of millions dollars each year [8]. Theoretically, preventing human rabies deaths by eliminating the disease in dogs incurs far lower cost than relying indefinitely on PEP to treat exposed individuals [9, 10].

To explore effective ways to control rabies in rural areas of Guangxi Province and create a model of rabies control that is applicable in rural China, we established a vaccination demonstration program for rabies prevention and control. Our aim was to vigorously promote rabies vaccination of domestic dogs in rural areas, to thereby significantly reduce the incidence of human rabies cases.

## Methods

### Information sources

Epidemiological data of human rabies in Guangxi Province from January 1982 to December 2017 were retrieved from the surveillance database of the National Disease Reporting Information System (NDRIS) of the Chinese Center for Disease Control and Prevention (CDC). According to the law of the People’s Republic of China on Prevention and Treatment of Infectious Diseases, all human rabies cases should be reported to the NDRIS within 24 h after diagnosis. Human rabies is diagnosed according to the National Diagnostic Criteria for Rabies (WS 281–2008).

### Vaccination program

The principle and basis of the present pilot vaccination program was that the incidence of human rabies is relatively high in China and the number of domestic dogs breeding in rural areas of the country is enormous. The demonstration program included a vaccination campaign, surveillance of clinically healthy dogs that might be carrying rabies virus (RABV), monitoring anti-RABV antibody titers in vaccinated dogs, and compiling and reporting statistics of human rabies cases.

### Vaccine

Before 2014, the rabies inactivated vaccine (Merial, France) was used for eradication program vaccination. In Guangxi Province, outside of the demonstration areas, attenuated RABV vaccine was used, which included ERA and HEP-Flurry strains. To improve the safety and immune effect, we stopped using attenuated vaccine in 2014 and switched to use of high-quality inactivated vaccine that includes SAD-B19 and CVS-11 strains, for vaccination across the entire province. All vaccines for the pilot project were provided free of charge by the Veterinary Medicine Department of the government of Guangxi.

### Rabies virus surveillance and virus isolation

From 1999 to 2017, brain samples were obtained from clinically healthy dogs (normal-looking dogs without rabies symptoms, including stray dogs, owned dogs, and dogs for sale commercially) in different regions of Guangxi Province, all brain samples collecting obtained informed consent from the dogs owners. Fluorescence assay (FA) was used to detect RABV nucleoprotein antigen, per WHO recommendations. RT-PCR was performed on the samples to detect RABV, and double positive samples were used for RABV isolation by mouse inoculation test [6]. Mice were purchased from the Animal Centre of Guangxi Medical University. To comply with Animal Research as Reporting In Vivo Experiments (ARRIVE) guidelines, all husbandry and experimental procedures were conducted in compliance with the Animal Welfare Act and the Guide for the Care and Use of Laboratory Animals. Mice were housed in temperature and light-controlled quarter, with access to food and water freely. The mice were euthanized in a container after application of halothane inhalant, with the container closed once the aenesthesized mouse displayed a lack of righting reflex (mouse unable to right itself within 10 s after being placed on its side).

Total viral RNA was extracted from brain samples using a TRIzol-based method (Invitrogen, Carlsbad, CA, USA), following the manufacturer’s instructions. cDNA was synthesized using 2.5 μg total RNA, 1 μL (25 pmol/mL) sense primer, and 100 U M-MLV reverse transcriptase (Promega, Madison, WI, USA) in a 25 μL reaction volume. RT-PCR amplification for diagnosis was carried out with primers RHN1 (5’-CTACAATGGATGCCGAC-3′) and RHN2 (5’-TTGCTCAACCTATACAGAC-3′) using Ex Taq DNA polymerase (Takara, China).

### Serological testing to detect seroconversion

Serum samples from vaccinated dogs in rural areas of different regions throughout Guangxi Province were randomly collected and prepared in the demonstration areas by animal disease control centers of local governments, all samples collecting obtained informed consent from the dogs owners. Serum anti-RABV antibody titers in vaccinated dogs were measured using ELISA kits (Synbiotics Europe SAS, Lyon, France). World Organisation for Animal Health (OIE) standard serum was provided by the OIE Reference Laboratory for Rabies (Changchun, China). The testing procedure was carried out according to the manufacturer’s instructions. The seroconversion rate of vaccinated dogs was determined using OIE standard serum as a reference. According to kit specifications, a positive antibody result to RABV was considered indicative of seroconversion.

### Statistical analysis

Initial data were analyzed using EpiData 3.1. Descriptive analyses were performed and presented using percentage or median and interquartile range to describe the demographic characteristics of cases. The relationship (r) tests and standard deviation (sd) were performed by Excell 2007.

## Results

### Epidemiological characteristics

**Fig. 1:**
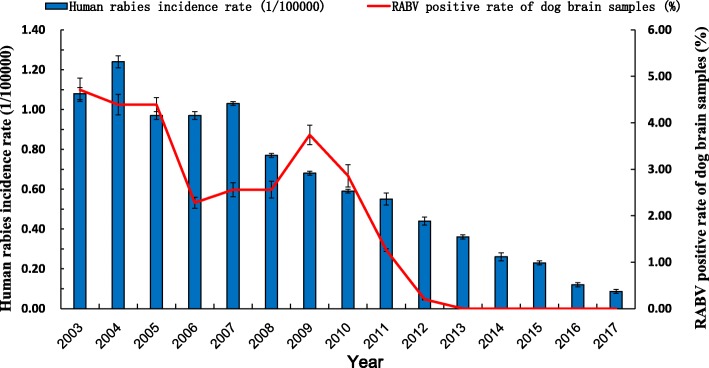
Comparison of the annual average incidence rate of human rabies and prevalence of clinically infected dogs in Guangxi Province

We evaluated the epidemiological characteristics of human rabies in Guangxi Province, using data of the NDRIS. A total of 10,225 human rabies deaths were reported in Guangxi in the 36 years from 1982 to 2017. The annual average incidence of the disease was 0.55 per 100,000 inhabitants. The annual incidence of human rabies displayed a decreasing trend from 2007 to 2017. The human rabies incidence rate (1/10,000) declined from 1.08 in 2007 to 0.09 in 2017, and the RABV-positive rate of dog brain samples declined from 3.74% in 2009 to 0 in 2017. There was a positive relationship between human incidence rate and RABV positive rate in dog brains (*r* = 0.889). The annual incidence rates of human rabies and RABV positive rate in dog brains during 2003–2017 are summarized in Fig. 1.

### Vaccination demonstration program

A wide range of dog vaccination campaigns against rabies were carried out in rural areas of China during 2003–2007, achieving a vaccination coverage rate of around 40%. To improve vaccination efficiency and accelerate the reduction in human morbidity, we implemented a pilot rabies vaccination program in Guangxi Province.

**Fig. 2:**
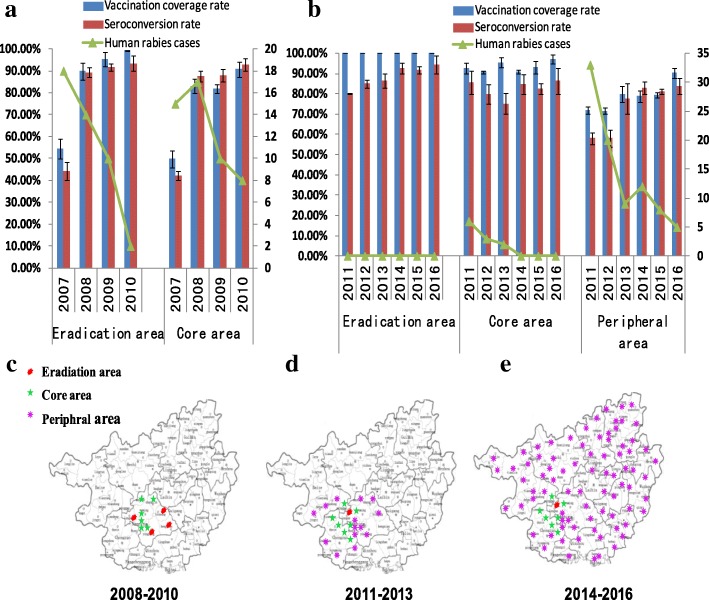
Pilot rabies vaccination program in Guangxi Province, by program phase, from 2008 to 2016. Annual average rates of dog vaccination, seroconversion, and human rabies, (**a**) by county in eradication area, and (**b**) by core and peripheral areas. **c**–**e** Location of eradication, core, and peripheral areas in each program stage

A total of 12 counties were divided into eradication and core areas in the first stage of the program, during 2008 to 2010. Of these, four counties (Binyang, Yongning, Longan, and Hengxian) were set as the eradication area; eight additional counties were set as the core area (Fig. 2c). In the eradication area, the average vaccination coverage rate of domestic dogs was 89.91, 95.26, and 99.30% each year from 2008 to 2010, respectively; the average seroconversion rate in these years was 89.38, 91.60, and 93.48%, respectively. The number of human rabies cases decreased from 18 cases in 2007 to 14, 10, and 2 cases each year from 2008 to 2010, respectively (Fig. 2a). In the core area, the average vaccination rate of domestic dogs reached 83.14, 81.86, and 90.78% each year from 2008 to 2010, respectively, and the average seroconversion rate was 87.50, 88.0, and 92.86%, respectively. There were 15 human rabies cases in 2007; this number decreased from 17 to 10 and then 8 cases each year from 2008 to 2010, respectively (Fig. 2a).

Based on the first stage, 21 counties were divided into eradication, core, and peripheral areas in the second stage from 2011 to 2013. Of these, 1 county (Longan) was set as the eradication area, 7 counties as the core area, and 13 counties as the peripheral area (Fig. 2d). In the eradication area, in each year during 2011 to 2013, the vaccination coverage rate reached 100% and the average seroconversion rate was 80.16, 85.06 and 86.56%, respectively; no human rabies cases occurred during the second stage of the project. In the core area, in each year during 2011 to 2013, the average dog vaccination rate in the 7 counties reached 92.59, 90.75, and 95.41%, respectively; the average seroconversion rate was 85.75, 79.92, and 75.08%, respectively; and the number of human rabies cases was 6, 3, and 2 cases, which was substantially lower than the 8 cases in 2010. In the peripheral areas, during 2011 to 2013, the average vaccination coverage rate in the 13 counties was 71.78, 71.65, and 79.91%, respectively, which was about 10% higher each year than that in 2010; the average seroconversion rate was 58.00, 58.00, and 77.50%, respectively; and the number of human rabies cases was 33, 20, and 9 cases (Fig. 2b).

**Fig. 3:**
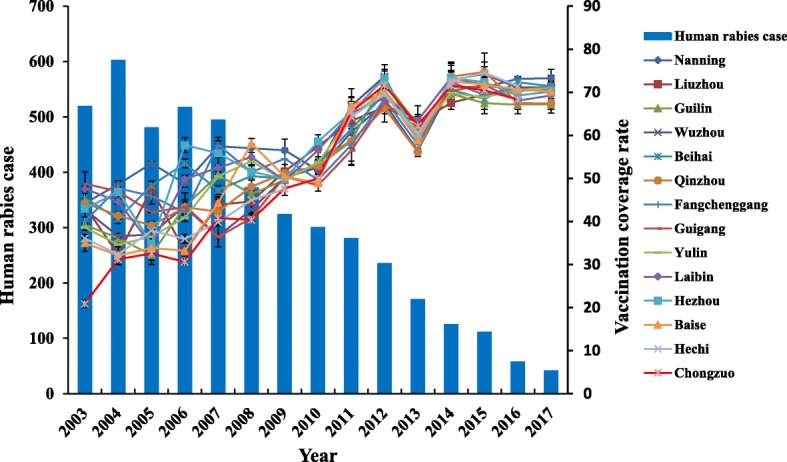
The increase in vaccination coverage rate of domestic dogs led to a decreased incidence of human rabies cases from 2003 to 2017

In 2014, the vaccination program model for rabies control was expanded and implemented in all 117 counties of Guangxi Province (Fig. 2e). Comprehensive statistical data and information on the effectiveness of the vaccination campaign were collected. Overall, the vaccination rate (Fig. 3) and average seroconversion rate (Fig. 2b) of domestic dogs was obviously increased in rural regions of Guangxi. From 2014 to 2017, a total 1,418,371, 1,357,045, 1,414,925, and 1,421,812 dogs were vaccinated each year, and the average dog vaccination rate in the whole of Guangxi Province reached 71.53, 71.17, 70.96, and 70.91%, respectively. A total of 1100, 1003, 528, and 553 dogs serum samples were collected randomly from different counties and tested for specific antibody each year. The average seroconversion rate was 82.55, 71.68, 79.92, and 96.20%, respectively. Therefore, the incidence of human rabies cases in the province declined sharply, there was a negative relationship between human rabies cases and dog vaccination coverage rate (*r* = − 0.954 to − 0.801) (Fig. 3).

### Surveillance of rabies virus in clinically healthy dogs

From 2003, surveillance has been carried out of clinically healthy dogs (normal-looking dogs with no rabies symptoms), to identify potential carriers of RABV. Since 2003, a total of 7083 brain samples have been collected from clinically healthy dogs in different regions of Guangxi Province and have undergone FA (Additional file 2: Figure S2) and RT-PCR, and positive samples by FA or RT-PCR were used for RABV isolation with a mouse inoculation test for detection of RABV. According to the results of detection, we found a positive correlation between human rabies incidence and RABV positivity rate of clinically healthy dogs (Fig. 1), suggesting that clinically healthy dogs have an important role in the spread of rabies from dogs to humans in rural regions.

## Discussion

Rabies vaccination on a large scale may be effective in reducing the RABV carrier rate among domestic dogs and may thus contribute to the decline of human rabies cases. Conversely, an approach that is based on reducing dog populations is unlikely to achieve stable effects [11]. Theoretical and empirical research has demonstrated that rabies could be eliminated where a 70% vaccination coverage is sustained [12, 13].

Rabies is highly prevalent in Guangxi Province, with more than 90% of human rabies cases occurring in rural regions. Domestic dogs have an important role in the transmission of rabies to humans in Guangxi, similar to other countries of Asia, Latin America, and Africa [14–16]. The principle reason is that the rabies vaccination rate among domestic dogs is low in rural areas, resulting in a large proportion of clinically healthy dogs that carry RABV; the link between dog bites and mortality risk in humans has been well recognized [7]. Rabies control in Latin America and the Caribbean has been successful, with certain approaches currently used, such as mass vaccination campaigns for dogs, postexposure prophylaxis, and epidemiologic surveillance [17]. Whereas there is widespread agreement about the central importance of mass dog vaccination in canine rabies control and elimination, the role of dog population management remains a subject of debate [17]. There is a rich body of literature concerning fertility control for management of roaming dog and wildlife populations [18]. Successful experiences of eliminating human rabies in Europe, Japan, the Caribbean, and Latin America have demonstrated promising methods for Guangxi to develop a more effective approach to controlling human rabies [19]. To explore effective ways of controlling this zoonosis in Guangxi Province according to the World Health Organization global framework for the elimination of dog-mediated human rabies, and to create an applicable model for controlling rabies in rural parts of China, a pilot rabies vaccination program based on the concept of One Health, which includes improvement of dog management and a special emphasis on the registration and restricted movement of domestic dogs, was established in the province as an active response to the WHO initiative.

The rabies vaccination demonstration program was carried out in three stages, with the coverage gradually expanded from 12 counties in the first stage to 21 counties in the second stage, and finally to the whole of Guangxi Province in the third stage. The program not only comprised monitoring of the dog vaccination rate, seroconversion rate, and surveillance of RABV-carrying clinically healthy dogs, it also included propaganda and technical training for the prevention and control of rabies. Between 2003 and 2016, once or twice per year in each county, a total 113,973 veterinarians, anti-epidemic personnel, and village committees in Guangxi Province were trained in rabies control and prevention. Through our great efforts in many areas, the vaccination coverage rate in Guangxi increased from about 40.68% before implementation of the campaign to 71.15% once the program was expanded to the entire province. In particular, the leaders of Longan County took dog vaccination program seriously and provided a lot of help in the vaccination campaign by organizing teams to individually check and register the number of dogs in each village and each family. This county was set as an eradication area from 2011 to 2016 and reached a dog vaccination coverage rate of 100%. Finally, the number of human rabies cases declined sharply from 602 cases in 2004 to 57 cases in 2016 and 41 cases in 2017 (Fig. 3). These results indicate that the vaccination demonstration program was highly effective in controlling rabies in Guangxi Province.

Human rabies remains a major public health problem in Guangxi, China [5]. To address this issue, interdisciplinary collaboration is required among veterinary services; departments of medicine, public health, and education; local authorities; and nongovernmental organizations [2, 20–22]. In addition, PEP remains an effective way to prevent rabies after a dog bite, despite its associated high economic burden. We implemented a pilot rabies vaccination program that included monitoring of the dog vaccination rate and seroconversion rate, as well as surveillance of potential carriers of RABV among clinically healthy dogs in Guangxi Province. Importantly, the number of human rabies cases declined substantially from 602 cases in 2004 to 41 cases in 2017. We will continue to explore approaches, such as those embodied within the principles of One Health, to achieve the aim of rabies eradication by further improving the effectiveness of vaccination efforts and improving dog management and registration in Guangxi Province, China.

## Conclusion

To explore effective ways to control rabies in rural areas of Guangxi Province and create a model of rabies control that is applicable in rural China, we established a vaccination demonstration program for rabies prevention and control.

The rabies vaccination demonstration program obviously increased rabies vaccination and seroconversion rates and effectively reduced the spread of rabies from dogs to humans, as well as the number of human rabies cases. The successful experience of the rabies vaccination demonstration program was applied to the entire province, thereby successfully controlling rabies in Guangxi, China.

## Additional files

Additional file 1: **Figure S1.** Location of Guangxi Province in China. (DOCX 150 kb)

Additional file 2: **Figure S2.** Fluorescence assay detection of rabies virus (RABV) in dog brain samples. Green fluorescence indicates RABV protein expressed in the cytoplasm. A, RABV-positive sample with fluorescence; B, RABV-negative sample. (DOCX 345 kb)

## Abbreviations

CDC: Center for Disease Control and Prevention
NDRIS: National Disease Reporting Information System
PEP: Postexposure prophylaxis
RABV: rabies virus
WHO: World Health Organization
