# Supplementary figures and images for: Vaccination demonstration zone successfully controls rabies in Guangxi Province, China
Source: BMC Infect Dis. 2018 Aug 10;18:386. doi: 10.1186/s12879-018-3301-8 (PMC6086044; doi:10.1186/s12879-018-3301-8)

A

B


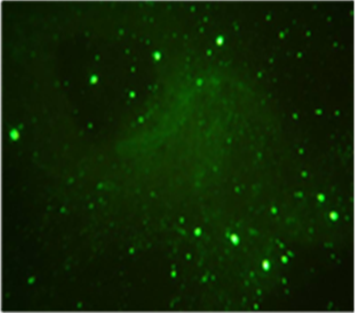

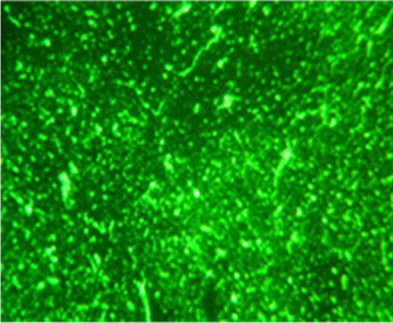


Figure S2

Supplement: Supplementary file 2 — Figure S2. Fluorescence assay detection of rabies virus (RABV) in dog brain samples. Green fluorescence indicates RABV protein expressed in the cytoplasm. A, RABV-positive sample with fluorescence; B, RABV-negative sample. (DOCX 345 kb) [file 12879_2018_3301_MOESM2_ESM.docx]
